# Breast Cancer Survivors' Perceptions of Their Cardiovascular Care During Treatment With Anthracyclines or Trastuzumab: A Qualitative Analysis

**DOI:** 10.1002/cam4.71106

**Published:** 2025-08-06

**Authors:** Arnethea L. Sutton, Ashley Turner, America Vijil, Victoria Williams, Paulette Omeaku, Wendy Bottinor, Avirup Guha, Lakeshia Cousin, Susan Hong, Christin Haynes, Sara Gómez‐Trillos

**Affiliations:** ^1^ Department of Kinesiology and Health Sciences Virginia Commonwealth University Richmond Virginia USA; ^2^ Massey Comprehensive Cancer Center Richmond Virginia USA; ^3^ Department of Health Policy Research Virginia Commonwealth University Richmond Virginia USA; ^4^ Division of Cardiology, Pauley Heart Center Virginia Commonwealth University Richmond Virginia USA; ^5^ Division of Cardiology, Department of Medicine Medical College of Georgia at Augusta University Augusta USA; ^6^ Cardio‐Oncology Program Medical College of Georgia at Augusta University Augusta Georgia USA; ^7^ Tampa General Hospital Tampa Florida USA; ^8^ College of Nursing University of South Florida Tampa Florida USA; ^9^ Division of Hematology, Oncology, and Palliative Care Virginia Commonwealth University Richmond Virginia USA; ^10^ Department of Social Work Virginia State University Petersburg Virginia USA; ^11^ Lombardi Comprehensive Cancer Center Georgetown University Medical Center Washington DC USA

**Keywords:** breast cancer, cardio‐oncology and cancer care delivery, cardiovascular disease, provider communication

## Abstract

**Background:**

Cardiovascular disease (CVD) is a leading cause of mortality among breast cancer survivors, and racial disparities exist between Black and White women. Therefore, the purpose of this study was to assess breast cancer survivors' perceptions of heart health resources and communication received during active treatment with potentially cardiotoxic therapies and to evaluate whether differences in perceptions are associated with race.

**Methods:**

This qualitative analysis included semi‐structured interviews with breast cancer survivors who received potentially cardiotoxic treatment(s). Survivors were asked to recall conversations with their healthcare providers regarding cancer treatment, the risk of CVD during and following treatment, and discussions of heart healthy behaviors that could be employed during treatment. Audio recorded interviews were transcribed, and codes were developed using Dedoose Qualitative Software.

**Results:**

Of the 17 participants, 9 women were White and 8 were Black. A majority of participants were married (58.8%), reported an annual household income of at least $60,000, had a Bachelor's degree or higher (70.6%), and had a mastectomy (70.6%). Participants had an average age of 50.9 years (SD = 11.85). Approximately 88% (100% White women, 75% Black women) recalled receiving heart health information either prior to or during treatment. The majority also received trastuzumab (88.2%). Four themes were identified from the interviews. Key results showed that Black survivors were more likely to share positive experiences when their family members were a part of their treatment conversations and that White women were more likely to have positive experiences throughout their treatment.

**Conclusions:**

This qualitative analysis did not show striking differences in perceived care experiences between Black and White survivors. However, there were notable differences in community support, such as the inclusion of family members in treatment conversations and the receipt and timing of heart health information. Enhancing healthcare communication with a particular focus on culturally diverse populations is needed.

**Abbreviations**
ACC: American College of Cardiology
AHA: American Heart Association
CVD: cardiovascular disease
EHR: electronic health record

## 1 Introduction

Breast cancer survivors are at increased risk of cardiovascular disease (CVD) following treatment when compared to women without breast cancer. Data have shown that older women with breast cancer are more likely to die from CVD than from breast cancer [1]. Further, racial disparities exist, as Black breast cancer survivors are up to three times more likely to develop and die of CVD when compared to their White counterparts [2, 3, 4, 5, 6]. The higher risk of CVD in breast cancer survivors is due, in part, to the receipt of potentially cardiotoxic treatments such as anthracycline chemotherapies and trastuzumab [2, 7]. Unfortunately, in a study by Williams et al., only 61% of breast cancer survivors in their cohort were aware that breast cancer survivors had an increased risk of CVD [8]. As the number of breast cancer survivors in the United States continues to rise [9], the need to identify ways to provide education and mitigate the risk of CVD becomes even more salient.

Various organizations provide patient‐facing guidance and resources to encourage cardiovascular health. Although not specific to cancer, the American Heart Association's Life's Essential 8 provides eight factors and measures needed to maintain cardiovascular health (e.g., get healthy sleep, manage weight) [10]. Specific to cancer survivors, the American College of Cardiology provides CardioSmart, and the International Cardio‐Oncology Society provides access to booklets and online videos [11, 12]. Existing evidence suggests that physical activity and exercise may mitigate the cardiovascular effects of treatment [13]. In spite of these resources, survivors' knowledge of their risk of CVD and its risk factors remains suboptimal. A recent study by Weaver and colleagues reported that within a cohort of breast cancer survivors, 65% were unaware of one or more of their CVD risk factors (i.e., blood glucose, cholesterol) and only 45% of survivors' cardiovascular health factors were determined to be ideal [14]. Few studies indicate a gap in CVD knowledge among breast cancer survivors, and none have sought to investigate the experiences and perceptions of cardiovascular care among breast cancer survivors who are at increased risk of CVD due to receipt of cardiotoxic treatments [15]. Additionally, to our knowledge, no studies have assessed the aforementioned areas in the context of racial disparities in CVD.

This phenomenological study used community engagement and qualitative approaches to examine breast cancer survivors' experiences receiving potentially cardiotoxic therapies during active breast cancer treatment. The purpose of this study was to (a) assess perceptions of provider communication about CVD during treatment, (b) identify survivors' approaches to ascertaining information about their cardiovascular health, and (c) explore racial differences in survivors' perceptions of provider communication about cardiovascular health.

## 2 Methods

### 2.1 Participants

Breast cancer survivors were recruited from a National Cancer Institute‐Designated Comprehensive Cancer Center in Richmond, Virginia. Eligibility criteria included those 18 years of age or older, identified as Black/African American or White, diagnosed with Stage 0–IV invasive breast cancer within the past 2 years, and having received anthracyclines and/or trastuzumab as part of their treatment. Leveraging the electronic health record (EHR), the cancer informatics core provided the principal investigator a list of potentially eligible women based on the aforementioned criteria. Study invitations, including the option to opt‐out, were emailed or mailed to women. Study participation required access to participants’ EHR and the completion of one semi‐structured interview. All study procedures were approved by the Virginia Commonwealth University Institutional Review Board. This study was granted exempt status; therefore, study participants received an information sheet prior to study enrollment.

### 2.2 Measures

Survivors responded to a brief questionnaire to collect data about their race, marital status, work status, education, and insurance status. This questionnaire was completed with a research assistant, and data were entered directly into REDCap, a secure web‐based data collection and management application. Clinical information, such as cancer stage and treatments, were abstracted from the EHR.

#### 2.2.1 Semi‐Structured Interviews

The interview guide was developed and refined in collaboration with two breast cancer patients and advocates who were familiar with the cardiotoxic potential of certain breast cancer treatments (Appendix S1 in Supporting Information S1). These partners ensured the use of appropriate terminology and reading level of the interview questions, that the order of the questions was logical, and that the questions encompassed the variety of provider types that care for cancer survivors during active treatment (e.g., oncologists, nurses). This partnership was integral to the success of the study, as these women leveraged their lived experiences to inform the appropriateness of the interview guide.

### 2.3 Procedures

The Principal Investigator (A.S., PhD) conducted one‐on‐one interviews with the survivors. At the time of the study, the interviewer, a female, was a postdoctoral fellow. The interviewer received informal (e.g., experiential learning) and formal (e.g., doctoral‐level course) training on qualitative methods, including data collection and analysis. Prior to conducting the interview, the interviewer had no prior relationships with the study participants. Study participants were informed of the goals of the research study and the overall goals of the interviewers' research program. The interviewer informed the participants of the reasons for the study, including the risk of and racial disparities in CVD following breast cancer treatments. The interviewer also collected field notes during the interviews. Since this study was conducted during the COVID‐19 pandemic, the interviewer conducted all interviews in a private home‐office.

The survivors were asked to recall the active treatment phase of their survivorship with the overarching theme of cardiovascular health resources and discussions. Questions explored the sources of cardiovascular health information, whether it was received from an oncologist or another member of the care team, and if participants sought cardiovascular health information from other sources or individuals. Participants were also asked about how they engaged in behaviors known to maintain cardiovascular health (e.g., exercise, health eating). For women who discussed receiving cardiac imaging, the interviewer asked questions about their experiences receiving surveillance. Participants could select to complete their interview either via phone or on Zoom. All interviews were audio‐recorded with the participant's permission and transcribed. Participants did not provide feedback on study findings, nor were their study transcripts returned to them. To fairly represent participants, exemplary quotes were given a deidentified letter. All participants received a $25 gift card for participating in the study.

### 2.4 Data Analysis

Univariate analysis in SPSS version 29.0 was used to describe the cohort (e.g., sociodemographic factors). Interviews were transcribed by Ubiqus on Demand, formerly Verbal Ink, and verified for accuracy by two research assistants. Following the Consensual Qualitative Analysis Framework, two research assistants (A.T. and A.V.), trained in qualitative data analysis, developed a codebook that was refined through multiple iterations. Data saturation, or the point when no new data were obtained from the interviews, was achieved after 17 interviews.

Most codes were developed deductively, based on the research questions and interview guides (Supporting Information 1). During the analysis process, inductive codes were added iteratively as new themes were identified. A.T. and A.V. independently coded all transcripts, and a third reviewer (P.O.) resolved any discrepancies. Dedoose Qualitative Software was used to code the interviews. Dedoose provides a collaborative platform so that all coders can organize the transcripts, abstract quotes, and identify and enumerate codes.

## 3 Results

### 3.1 Sociodemographic and Clinical Factors

Participants (*N* = 17) had an average age of 50.9 years (standard deviation ± 11.85). Nine women were White and eight were Black (Table 1). A majority of participants were married (58.8%), reported an annual household income of at least $60,000, had a Bachelor's degree or higher (70.6%), and had a mastectomy (70.6%). The majority also received trastuzumab (88.2%).

**TABLE 1:** Breast cancer survivors' characteristics (*n* = 17).

Variable	All women *N* (%)	Black women *N* (%)	White women *N* (%)
Age: mean (SD)	50.9 (11.85)	46.1 (10.56)	55.1 (11.93)
Race			
Black	8 (47.1%)	—	—
White	9 (52.9%)		
Marital status			
Married Divorced	10 (58.8%) 2 (11.8%)	3 (37.5%) 1 (12.5%)	7 (77.8%) 1 (11.1%)
Single (never married)	5 (29.4%)	4 (50%)	1 (11.1%)
Total household income, $			
< $60,000	8 (47.1%)	4 (50%)	4 (44.4%)
≥ $60,000	9 (52.9%)	4 (50%)	5 (55.6%)
Education			
12th grade or GED	2 (11.8%)	2 (25%)	0 (0%)
Some college	1 (5.9%)	0 (0%)	1 (11%)
Bachelor's degree or higher	12 (70.5%)	5 (62.5%)	7 (77.8%)
Other than college (e.g., technical or vocational training)	2 (11.8%)	1 (12.5%)	1 (11.1%)
Employment status			
Full‐time	5 (29.4%)	3 (37.5%)	2 (22.2%)
Part‐time	1 (5.9%)	0	1 (11.1%)
Retired	4 (23.5%)	1 (12.5%)	3 (33.3%)
Unemployed (seeking employment)	3 (17.7%)	2 (25%)	1 (11.1%)
Other (e.g., disabled and self‐employed)	4 (23.5%)	2 (25%)	2 (22.2%)
Surgery type			
Mastectomy	12 (70.6%)	7 (87.5%)	5 (55.6%)
Lumpectomy	4 (23.5%)	1 (12.5%)	3 (33.3%)
No surgery	1 (5.9%)		1 (11.1%)
Radiation			
Yes	9 (52.9%)	4 (50%)	5 (55.6%)
No	8 (47.1%)	4 (50%)	4 (44.4%)
Endocrine therapy			
Yes	8 (47.1%)	5 (62.5%)	3 (33.3%)
No	9 (52.9%)	3 (37.5%)	6 (66.7%)
Cancer stage			
I	5 (29.4%)	2 (25%)	3 (33.3%)
II	6 (35.3%)	4 (50%)	2 (22.2%)
III	4 (23.5%)	1 (12.5%)	3 (33.3%)
IV	2 (11.8%)	1 (12.5%)	1 (11.1%)
Heart issues prior to breast cancer diagnosis			
Yes	2 (11.8%)	1 (12.5%)	1 (11.1%)
No	15 (88.2%)	7 (87.5%)	8 (88.9%)
Time since diagnosis (months): mean (SD)	29.4 (9.9)	31.0 (9.12)	28.0 (11.39)

Four themes were identified from the interviews: (1) Positive assessment of care during receipt of cardiotoxic treatment; (2) Negative assessment of care during receipt of cardiotoxic treatment; (3) Assessments of heart health information; and (4) Survivor recommendations to improve heart health communication and care during survivorship. To fairly represent participants, exemplary quotes were given a deidentified letter (Table 2).

**TABLE 2:** Breast cancer survivors' direct quotes by theme.

Positive assessment of care during receipt of cardiotoxic treatment
*White participants*
Participant K: “I mean, the heart could still get radiation, okay, but he explained all that to me and went over everything, and felt very prepared for my radiation. The radiation technologists were just excellent”
Participant J: “I actually just wrote [Oncologists Name] up for an award, just because I – it's hard for somebody to be newly diagnosed and come in on that first day, and leave understanding what's going to happen, because you're so overwhelmed… And I left being like, okay, I literally don't have a question”
Participant H: “I also wanna say that the nurses there in the transfusion center, they are your dearest friends during this. I couldn't even tell you always their names. I knew their faces, and for a while I knew their names. I don't know where they live or whether they have kids or not. I'm just saying that they are such good caregivers”
*Black participants*
Participant N: So, yeah, I had a lot of family support and my daughter, she's a registered nurse. So, you know, she knew a lot of the questions to ask, some of the things that I should be concerned about. So, [Oncologists Name], she welcomed all of that… And what you don't always find, sometimes doctors are very—they don't want to get into the family stuff. But that was very important to me that they support my family
Participant P: They were outstanding. You know, they came up with a plan to where—what's going to happen and how we expect it to happen, you know?
Participant M: I'm going to speak highly of her, the oncologist and my surgeon. They didn't cut any corners with me. They told me everything, all the information that I needed to know. And if I didn't know, I asked the question or whatever, like that

Negative assessment of care during receipt of cardiotoxic treatment
*White participants*
Participant E I didn't really like him, because he was—he [imaging tech] talked very medical terminology to me and I'm like, “Huh?”
Participant I: I felt overwhelmed. And what else? There was—this is going to sound absolutely crazy but my radiation oncologist, a really nice man. He did not always make me feel comfortable
Participant H: They don't really tell you what they're looking for, what they're looking at. You just are there for a long time, but it's very uncomfortable. I mean, they're pressing on things that are still kind of sore, and I just think a little heads‐up notice would have been—or maybe somebody a little bit more attuned to what you'd been through
*Black participants*
Participant O: The scheduler called, set it up. I went to, you know, the location, had it done. It was very stressful, I will say, during the actual 30‐minute time period that it took. And then to know that, again, I have to wait three to five days to have it read
Participant C: But like if I'm in a moment or just having an open door is better than just, I'm available when I'm available. She wasn't really—she wasn't there the way they made it seem
Participant L: And sometimes, I felt like I was just like a patient number—like a stat—as opposed to a person, and then, because of that, I did not feel comfortable. I did not feel like I could relate especially to the surgeon. Like, for example, I start getting very sensitive to little things like, “Okay. Is he treating me like a person or just like patient number so and so coming?

Assessments of heart health information
*White participants*
Participant B: Well, the fact that they told me if I had any issues [Heart Problems] that they would either take me off of it or that they would monitor it. So I felt comfortable with it [treatment]
Participant J: “I don't recall. I know we did ECHOs so we could check… So yes, they must have”
Participant F: [Oncologist] said something like for as long as you had been two years on a [monoclonal antibody] and [trastuzumab] it wasn't such a good idea because it can damage the heart. The longer that you stay on it will damage the heart. And she made the decision to stop it
*Black participants*
Participant N: Well, I think during the whole process, they were concerned about my heart mainly. You know, but there were no problems
Participant D: I know my oncologist talked to me about that too. That's the whole goal to try to keep it from coming back. She did say that it can—she also did talk to me about my heart. Because when I had the other—when I had my first round of chemo, my heart function was 45. So I couldn't do any treatments or do the [trastuzumab] until my heart function got back to normal
Participant M: They told me—and prior to everything, they told me as far as the burning of the skin, and as far as it affecting my heart, they explained to me well, you know, the side effects

Survivor recommendations to improve heart health communication and care during survivorship
*White participants*
Participant H: “They're treating the cancer. I get that. But I was still kinda surprised at the one note approach that my oncologist has, and no offense. She was recommended by a good friend of mine. She's like “She's the best. You're gonna love her” and I do feel like she was on top of the whole cancer thing, but as far as taking care of the person, no way”
Participant K: I think maybe a little bit more emphasis on heart, simply what can happen… I think more heart discussions would be—what to look for, what to report so that they don't think you're a neurotic person now
Participant Q: I think it needs to be parsed out um, you know, in a way that it's, it's retained. Because there's so much that they tell you in the beginning that you're just like, what? And I think if it's like another thing you're like okay, I'm out. I just, I can't even process all this. Um, so maybe you know, not in the beginning.
*Black participants*
Participant G: I think that having that person [Cardiologists] on the team it could possibly be a scary thing but at least you have somebody that's following it
Participant O: I think one thing that would be important is get the—get the lady on a regiment like…to look at it, “Okay. This person has been through chemo last year. Let's reach back out to her and let's go ahead and do another EKG just to see where she is now
Participant D: Yes, or I wished they would have emphasized it [exercising] more and then even say, hey—I think it would help patients… but if the way they scheduled tests were, well, we need you to take this test [exercise]

### 3.2 Positive Assessment of Care During Receipt of Cardiotoxic Treatment

Of the sample, 78% of White women reported positive experiences with their healthcare provider, compared to 50% of Black women. Participants appreciated provider communication that adequately prepared them for their treatment. For example, participant K (White woman) stated:

I mean, the heart could still get radiation, okay, but he [physician] explained all that to me and went over everything, and I felt very prepared for my radiation. The radiation technologists were just excellent.

Participants reported being satisfied when all of their questions about their treatment plan were answered, or similarly, when they left their appointments feeling that they had no questions to ask. While both Black and White women reported positive assessment, one difference noted was that 25% (*n* = 2) of Black participants preferred familial support compared to 11% (*n* = 1) of white participants. Regarding specific healthcare professionals, both Black and White women reported that nurses were the main facilitators of heart health communication. Although participants discussed communication with their physicians (e.g., oncologists), most information was provided by nurses.

Participants also discussed positive experiences during their imaging. For example, participant K stated that during ultrasound,

They would start doing…the examination and everything. I would always be scared or something. They [ultrasound tech] would always make it comfortable for me. We would always talk about something positive.

### 3.3 Negative Assessment of Care During Cardiotoxic Treatment

Among the sample, seven Black participants (63%) had a negative experience with their healthcare provider compared to four White participants (43%). A majority of Black participants discussed bedside manner, particularly related to the use of technical jargon when discussing the process of cardiac testing/imaging and cardiac imaging results. One participant reported that she told her oncologist she did not want to see the provider in cardiology again for this reason. Similarly, another participant voiced frustration about not receiving their cardiac imaging in real‐time: participant E reported

I didn't really like him, because he was—he talked very medical terminology to me and I'm like, ‘Huh?’ So I didn't know what he was talking about… I told [Oncologists], I said, ‘I don't want to go to him again.’ I said, ‘I need somebody that can talk to me in layman's terms’.

Although only reported by one participant, it is worth noting survivors' perspectives on the ways in which cardiotoxic treatments are named, as participant G reported,

I was told that it was the red devil. And I said no ma'am. I blocked it out immediately because it was shocking to hear her say that and it was disappointing at the same time because when you are fighting for your life, you don't know what any outcome is going to be.

### 3.4 Assessments of Heart Health Information

Every White woman in the study recalled receiving heart health information either prior to or during treatment; 75% of Black women stated they received heart health information. Two women who received trastuzumab discussed ejection fraction, without being prompted. For example, one White woman stated,

I think my ejection fraction stayed where it needed to be…there was a threshold and I was informed that as long as you are above, I believe it was 50, something like that, then you are okay.

Of the women who recalled receiving heart health information, many stated their providers discussed different food options to incorporate into their daily lives and provided guidance on where to find additional information about a healthy diet and exercise. Fourteen women (87.5%) reported implementing health behaviors during treatment following a discussion with their healthcare provider. White survivors (vs. Black) were more likely to report exercising and eating healthy during treatment. Four women received written resources, such as pamphlets, that discussed diet and exercise. However, two of the women who received these resources, stated they did not read or further research the topic.

### 3.5 Survivor Recommendations to Improve Heart Health Communication and Care During Survivorship

Some participants provided spontaneous suggestions about care provision overall and specific to cardiovascular health. It was observed that Black participants were not only more likely to provide suggestions in general, but they were also more likely to suggest receipt of or improvements in heart health information when compared to White participants. For example, Participant D (Black woman) stated:

Yes, or I wished they would have emphasized it [exercising] more and then even say, hey– I think it would help patients… but if the way they scheduled tests were, well, we need you to take this test [exercise].

Participants also alluded to the need for more cardiovascular care and surveillance following active treatment:

I think to keep it a priority would be that the doctors are having constant conversations about your heart…because from what I understand that there is this problem could arise at any time during the first five years or something like that with your heart – I guess or even later. (Participant N—Black Woman)

Interestingly, only one woman recommended, based on her personal preferences, that providers not discuss heart health side effects of treatment as she felt that it may have initially deterred her from receiving the treatment. It is also important to note that this woman did not recall receiving any information about the potential impact of the treatment on her heart.

## 4 Discussion

This qualitative inquiry is one of the first to provide breast cancer survivors' perceptions of the information they received regarding heart health during active breast cancer treatment. Although survivors reported varying levels of overwhelm with the amount and types of information they received upon and during their breast cancer diagnosis, our findings highlight survivors' desires to receive information about cardiovascular health throughout the survivorship journey. While this study did not uncover stark disparities in care experiences between Black and White survivors, there were a few noteworthy differences reported, including the inclusion of family members in treatment conversations and the receipt and timing of heart health information. Our findings provide valuable insight into the role of provider communication by specifically examining how differences in provider interactions influence survivors' perceptions of care—these interactions may impact women's participation in heart healthy behaviors.

White and Black women reported positive and negative assessments of communications, but positive experiences were more frequently reported by White women, while more negative experiences were reported by Black survivors. This finding may be due to various factors. Studies have reported similar racial differences in the assessments of provider communication during cancer treatment [16, 17, 18]. Interestingly, regarding healthcare decision‐making, data show differences in provider communication by race. For example, Sheppard et al. reported that greater provider communication was significantly associated with receipt of chemotherapy in Black breast cancer survivors, but lesser provider communication was associated with uptake in White survivors [19]. A study investigating the role of provider communication on the uptake of cancer screenings reported racial and ethnic differences in provider communication preferences (e.g., teach‐back, patient‐centered communication) [20]. Given the salient role of provider communication and interactions on survivors' decisions to implement risk reduction behaviors, future efforts should incorporate culturally tailored and patient‐centered communication that incorporates survivors' needs, values, and preferences.

Survivors desired more information about exercising during treatment. This is a particularly interesting area, as emerging studies highlight the potential positive impact of exercise on cardiovascular health during and following active cancer treatment. A recent study by Bellissimo et al. reported that physical activity attenuated declines in cardiovascular health (e.g., exercise capacity) in breast cancer survivors who were undergoing active treatment [21]. While such findings provide hope, studies also demonstrate that Black breast cancer survivors present with poorer cardiovascular health prior to cancer treatment, and one recent study found that when compared to White breast cancer survivors, Black survivors experienced a greater reduction in exercise capacity, a measure of cardiovascular health, 3 months following treatment [22]. As data continue to emerge on the benefits of exercise and physical activity during treatment, effective and innovative approaches are warranted to educate, encourage, and support breast cancer survivors' engagement in heart health behaviors during treatment.

## 5 Limitations

Strengths of this study include the close to equal representation of Black and White breast cancer survivors, and survivor‐reported recommendations for care and communication improvements. While our study provides valuable insights into the knowledge gaps surrounding essential heart‐healthy communication following breast cancer treatments, several limitations should be considered when interpreting these findings. During the semi‐structured interviews, survivors were asked to recall from memory their experiences and perceptions of provider communication regarding their treatment. Recall bias was possible as some survivors could have been up to 2 years from their diagnosis. While we discussed survivors' experiences with their providers, we did not collect data on the providers' race. This information may have provided additional context, particularly regarding racial concordance/discordance and perceptions of provider communication. Lastly, while Black breast cancer survivors do have higher rates of CVD than their White counterparts, it is important to consider that those with very positive experiences in care may have been more inclined to participate than those who received less satisfactory care. Additionally, Black survivors in this sample had a higher prevalence of mastectomy than White survivors, which, conversely, may have contributed to the higher negative communication ratings given the perceived enhanced treatment complexity of mastectomy when compared to lumpectomy. These potential selection biases were acknowledged in our analysis, and we interpreted the findings with an understanding that they might represent more extreme experiences.

## 6 Conclusions

Our study findings indicate that Black survivors' may value integrating their social networks in their care discussions and a need for healthcare providers to receive further training in culturally sensitive communication surrounding CVD preventative behaviors. Additional training should focus on how to effectively communicate cardiovascular risks to breast cancer survivors, considering the emotional and informational needs of diverse populations. Further research should investigate how well survivorship care plans are implemented in practice, particularly regarding the inclusion of cardiovascular health information. Further, additional exploration into how the quality of provider communication impacts long‐term cardiovascular health outcomes among breast cancer survivors. By addressing these gaps, our understanding of survivorship care is not only enhanced but can lead to the development of targeted interventions that can improve cardiovascular outcomes and overall quality of life for breast cancer survivors.

## Author Contributions

**Arnethea L. Sutton:** conceptualization (lead), data curation (lead), formal analysis (equal), funding acquisition (lead), investigation (lead), methodology (lead), project administration (lead), resources (lead), software (equal), supervision (lead), validation (equal), visualization (equal), writing – original draft (lead), writing – review and editing (lead). **Ashley Turner:** conceptualization (lead), data curation (lead), formal analysis (lead), investigation (equal), methodology (equal), project administration (lead), resources (equal), software (equal), supervision (equal), validation (equal), visualization (equal), writing – original draft (lead), writing – review and editing (lead). **America Vijil:** conceptualization (supporting), data curation (equal), formal analysis (equal), funding acquisition (supporting), investigation (supporting), methodology (supporting), project administration (supporting), resources (supporting), software (supporting), supervision (supporting), validation (supporting), visualization (supporting), writing – original draft (supporting), writing – review and editing (supporting). **Victoria Williams:** conceptualization (supporting), data curation (supporting), formal analysis (supporting), investigation (supporting), methodology (supporting), project administration (lead), resources (supporting), software (supporting), supervision (supporting), validation (supporting), visualization (supporting), writing – original draft (supporting), writing – review and editing (supporting). **Paulette Omeaku:** conceptualization (supporting), data curation (equal), formal analysis (equal), funding acquisition (supporting), investigation (supporting), methodology (supporting), project administration (supporting), resources (supporting), software (supporting), supervision (supporting), validation (supporting), visualization (supporting), writing – original draft (equal), writing – review and editing (equal). **Wendy Bottinor:** conceptualization (supporting), data curation (supporting), formal analysis (supporting), funding acquisition (supporting), investigation (supporting), methodology (supporting), project administration (supporting), resources (supporting), software (supporting), supervision (supporting), validation (supporting), visualization (supporting), writing – original draft (supporting), writing – review and editing (supporting). **Avirup Guha:** conceptualization (supporting), funding acquisition (supporting), investigation (supporting), methodology (supporting), resources (supporting), validation (supporting), visualization (supporting), writing – original draft (supporting), writing – review and editing (supporting). **Lakeshia Cousin:** conceptualization (supporting), formal analysis (supporting), investigation (supporting), methodology (supporting), resources (supporting), validation (supporting), writing – original draft (supporting), writing – review and editing (supporting). **Susan Hong:** conceptualization (supporting), investigation (supporting), methodology (supporting), resources (supporting), writing – original draft (supporting), writing – review and editing (supporting). **Christin Haynes:** conceptualization (supporting), investigation (supporting), resources (supporting), writing – original draft (supporting), writing – review and editing (supporting). **Sara Gómez‐Trillos:** conceptualization (supporting), data curation (supporting), formal analysis (supporting), investigation (supporting), methodology (supporting), resources (supporting), supervision (supporting), writing – original draft (supporting), writing – review and editing (supporting).

## Disclosure

The authors have nothing to report.

## Consent

Signed informed consent was not required, as this study was granted exempt status by the IRB. Study participants did, however, receive a study information sheet prior to enrollment.

## Conflicts of Interest

The authors declare no conflicts of interest.

## Supporting information


**Data S1:** cam471106‐sup‐0001‐Supinfo.docx.

## Data Availability

Data archiving is not mandated but will be made available on reasonable request.
