# Supplementary material for: Breast Cancer Survivors' Perceptions of Their Cardiovascular Care During Treatment With Anthracyclines or Trastuzumab: A Qualitative Analysis
Source: Cancer Med. 2025 Aug 6;14(15):e71106. doi: 10.1002/cam4.71106 (PMC12326079; doi:10.1002/cam4.71106)
Supplement: Supplementary file 1 — Data S1: cam471106‐sup‐0001‐Supinfo.docx. [file CAM4-14-e71106-s001.docx]

**Supporting Information S1:** **Interview Guide Questions**

**Introduction:**

The purpose of this interview today is to understand more about your experience

receiving treatment for your breast cancer. We are going focus on you experiences receiving

anthracycline chemotherapy or Herceptin. We are also interested in the heart health of breast

cancer survivors who receive the type of treatment(s) you received. I hope that you will

candidly share your thoughts, feelings, or concerns with me. Please know that all information

that you share is confidential. We will not use any names of individuals in any of the material. If at any time you would need to stop, please let me know. As we discussed when you consented,

I will be audio recording this interview.

Before I start the recording, do you have any questions for me?

1. According to our records, you received [anthracycline chemotherapy and/or Herceptin]

for your breast cancer treatment. Tell me a little about your experience with your

treatment.

1. Did your oncologist or another provider talk to you about potential side effects of that

treatment and what were they?

- Probe: Yes or NO: Did you hear of the side effects from other sources? If so, what were they?

1. Did your oncologist talk to you about how your treatment may impact your heart?

- Probe: If yes, what were your feelings about that discussion and did it impact your decision to receive that treatment? If not, would you have liked to know of that potential side effect? Why do you believe your provider did not discuss that with you?

1. Did your doctor check your heart during your treatment? What was that

process like?

- Probe: How did you feel during those evaluations?

1. Did your doctor ever tell you that they were concerned or saw issues with your heart

while on your treatment?

- Probe: If yes, how did that make you feel? Did you share this information with anyone?

1. Did your doctor talk to you about ways to stay as healthy as possible during your treatment? Did they offer you any tips to try to protect your heart?

- Probe: If yes, did you try any of those tips and who did you feel?

1. Did you seek out information about heart health during your treatment from other places, like websites, or other people?
2. Now that you are [# of years] out from your treatment, what are your feelings or

thoughts about your heart health now?

1. Is there anything else about your heart or treatment that you would like to discuss or

that you feel it is important for us to think about when we talk to other women?

**Closing:**

Thank you very much for taking time to talk with me. Please confirm the mailing

address where you would like to receive your gift card.

**Appendix S2:** **Codebook/Open Codes**

| **CFIR Domain** | **Axial Code** | **Open Codes** |
| --- | --- | --- |
| Heart Health | Experiences with side effects related to heart health | Experiences with side effects related to heart health, both at the time of diagnosis and at the time of the interview. |
|  | Information about heart health | Information from providers about heart health risk and information regarding concerns.  Information-seeking behavior from other sources, such as websites, friends, family, nutritionists, cardiologists, etc.  The patient's feelings about this information. |
|  | Feeling about treatment plan | The patient's feelings during these evaluations  Both at the time of treatment and at the time of the interview. |
|  | Monitoring and treatment plan specific to heart health | Patient communicating their treatment plan in regards to their heart health. |
| Experiences while on treatment | Specific to chemotherapy | Patients lived experiences specifically related to them receiving chemotherapy. |
|  | Specific to Herceptin | Patients lived experiences specifically related to them receiving Herceptin. |
|  | Overlap of chemotherapy and Herceptin or unsure | Patients lived experiences related to them receiving chemotherapy and Herceptin at the same time or when the causal treatment cannot be distinguished. |
|  | Helpful Coping Strategies | Patient’s perceived usefulness of different strategies they used to mitigate side effects, including, but not limited to, adopting the protective behaviors recommendations by providers. |
|  | Long Term side effects | Patients experiencing long term side effects (1 year post treatment, not including endocrine/hormonal therapy) after treatment. |
| Communication with healthcare providers | With MDs | Patient’s experiences communicating with MDs, including their oncologists, cardiologists, nutritionists, feelings about the communication, their positive and negative experiences, topics of communication, and suggestions for improvement. |
|  | With NPs | Patient’s experiences communicating with nurses and nurse practitioners about the communication, their positive and negative experiences, topics of communication, and suggestions for improvement. |
|  | With other providers (Nutritionist, dietitian, nurses, or medical assistants) | Patient’s experiences communicating with other healthcare providers about the communication, their positive and negative experiences, topics of communication, and suggestions for improvement. |
|  | General information | Information and resources outside of heart health provided by healthcare providers. |
|  | Communication about side effects | Healthcare provider and patient discussing side effects. |
|  | Protective behavior | Healthcare provider providing coping strategies and tips to mitigate adverse reactions. |
|  | Positive experiences | Patients' positive experiences while communicating with their healthcare provider. |
|  | Negative experiences | Patients' negative experiences while communicating with their healthcare provider. |
|  | Suggestions for improvement | Patients expressing their overall experiences of their treatment plan including provider-patient communication, communication about possible side effects, and communication about disease. |
| Heart Health Preferences | Discussing Heart health during treatment | Patients communicate heart health **during treatment** with any provider. |
|  | Understanding of heart health information | The patients comprehension of heart health that was given by any provider. |
| Outside health resources |  | Patient seeking **non heart health information** outside of assigned healthcare provider. For example websites, social media, friends and family. |
